# *Candida albicans *induces cyclo-oxygenase 2 expression and prostaglandin E2 production in synovial fibroblasts through an extracellular-regulated kinase 1/2 dependent pathway

**DOI:** 10.1186/ar2661

**Published:** 2009-03-29

**Authors:** Herng-Sheng Lee, Chung-Shinn Lee, Chi-Jung Yang, Sui-Long Su, Donald M Salter

**Affiliations:** 1 Department of Pathology, Tri-Service General Hospital and National Defense Medical Center, No. 325, Sec. 2, Chenggong Rd, Neihu District, Taipei City 114, Taiwan; 2 Graduate Institute of Pathology and Parasitology, National Defense Medical Center, No. 161, Minchun E. Rd, Neihu District, Taipei City 114, Taiwan; 3 School of Public Health, National Defense Medical Center, No. 161, Minchun E. Rd, Neihu District, Taipei City 114, Taiwan; 4 Osteoarticular Research Group, Centre for Inflammation Research, Queens Medical Research Institute, 47 Little France Crescent, Edinburgh, EH16 4TJ, UK

## Abstract

**Introduction:**

Synovial cells are potential sources of inflammatory mediators in bacterial-induced arthritis but their involvement in the inflammatory response to *Candida albicans*-induced septic arthritis is largely unknown.

**Methods:**

Primary cultures of rat synovial fibroblasts were infected with *C. albicans *(ATCC90028). Immunocytochemistry, western blotting, and RT-PCR were performed to assess cyclo-oxygenase 2 induction. Phosphorylation of extracellular-regulated kinase (ERK1/2) following infection in the absence or presence of U0126 was assessed by western blotting whilst prostaglandin E2 production was measured by ELISA. Nuclear factor κB (NFκB) translocation was evaluated by an electrophoretic mobility shift assay.

**Results:**

Infection of synovial fibroblasts with *C. albicans *resulted in cyclo-oxygenase 2 expression and prostaglandin E2 production. Cyclo-oxygenase 2 expression and prostaglandin E2 production was dependent upon extracellular-regulated kinase 1/2 phosphorylation, associated with activation of NFκB and significantly elevated in the presence of laminarin, an inhibitor of dectin-1 activity. Synovial fibroblasts adjacent to *C. albicans *hyphae aggregates appeared to be the major contributors to the increased levels of cyclo-oxygenase 2 and phosphorylated extracellular-regulated kinase 1/2.

**Conclusions:**

*C. albicans *infection of synovial fibroblasts *in vitro *results in upregulation of cyclo-oxygenase 2 and prostaglandin E2 by mechanisms that may involve activation of extracellular-regulated kinase 1/2 and are associated with NFκB activation.

## Introduction

Infectious arthritis is a potentially serious disease that may cause rapid destruction of the joint and produce permanent deformities. Articular structures can be affected by mycotic infections through direct inoculation, contiguous spread, or hematogenous dissemination [1-4]. Of the various Candida species, *Candida albicans *is most commonly associated with fungal arthritis, especially in immunocompromized individuals [4-7]. Typically infection predominates in large weight-bearing joints, most often the knee [8]. Experimental arthritis in Sprague-Dawley rats with intravenous administration of *C. albicans *demonstrates that Candida arthritis involves not only joint tissues but also adjacent bones [9]. In mice, direct inoculation of joints with *C. albicans *results in a rapidly progressive septic arthritis that also exacerbates collagen-induced arthritis [10]. Fungal infection may also induce and exacerbate autoimmune diseases such as rheumatoid arthritis potentially through effects of β-glucans, polysaccharides in the cell wall of fungi, on inflammatory and immune responses [11].

Cyclo-oxygenase 2 (COX-2) is a key enzyme involved in joint inflammation through production of prostaglandins. COX-2 is induced in human joint tissues, including chondrocytes and synoviocytes, by inflammatory stimuli such as interleukin 1β (IL1β), IL17, and tumor necrosis factor (TNF) [12-20] and has roles in cartilage degradation and synovial angiogenesis [16,21]. Micro-oganisms of all types, mostly bacterial infections, can produce an infectious arthritis associated with COX-2 induction and prostaglandin E_2 _(PGE_2_) production [22-24]. In response to *C. albicans *infection HeLa cells [25], vascular endothelial cells [26], and macrophages *in vitro *[27] have been shown to express COX-2. The signal transduction pathways resulting in COX-2 expression may involve Toll-like receptor (TLR) 2 and 4 [25,28], which activate a variety of signaling molecules including p38 [29], c-Jun N-terminal kinase (JNK) [29,30], extracellular-regulated kinase (ERK) [31,32], protein kinase C (PKC), and activated nuclear factor κB (NFκB) [25,30]. More recently dectin-1 the receptor for β-glucan a fungal wall component has been shown to be involved in the induction of cytokines and chemokines possibly by collaborating with TLRs [33].

Although it is well documented that *C. albicans *may induce joint inflammation and destruction, the detailed inflammatory responses and associated mechanisms are largely unknown. The present study was undertaken to establish a model to examine COX-2 induction in synovial fibroblasts following *C. albicans *infection *in vitro*.

## Materials and methods

### Synovial fibroblast isolation and culture

Male Sprague-Dawley (SD) rats (8 weeks old, 280 to 300 g) were obtained from BioLASCO Taiwan (Taipei, Taiwan). All experiments were approved by the local Institutional Review Board and performed in adherence to the National Institutes of Health Guidelines for the treatment of laboratory animals. The synovium of knee joints was aseptically removed from normal SD rats, cut into small fragments and incubated with antimicrobial solution (500 IU/ml penicillin/streptomycin; Gibco Invitrogen, Burlington, Ontario, Canada) for 1 h, washed with sterile phosphate-buffered saline (PBS) before digestion with 3 mg/ml collagenase type H (Sigma, St Louis, MO, USA) at 37°C for 12 h. The resultant cell suspension was centrifuged at 2,500 rpm for 10 minutes following which the supernatant was discarded and the pellet resuspended in PBS. After further centrifugation at 1,000 rpm for 10 minutes, cells were resuspended and seeded in 20 ml of Ham's F12 medium (Sigma) containing 10% fetal bovine serum (Gibco Invitrogen) and 100 IU/ml penicillin/streptomycin (Gibco Invitrogen). The synovial cells were then cultured in a humidified 5% CO_2 _atmosphere at 37°C until confluent, detached with 0.05% trypsin/ethylenediaminetetraacetic acid (EDTA) (Gibco Invitrogen) and seeded at a density of 2 × 10^5 ^cells/dish in 60 mm tissue culture dishes (Orange scientific, Braine-l'Alleud, Belgium) for further experimental procedures.

### *C. albicans *preparation

*C. albicans *(ATCC 90028) was grown on Sabouraud dextrose agar (BD Microbiology System, Sparks, MD, USA) at 25°C. After a 16-h culture, colonies were suspended in PBS (Gibco Invitrogen) and prepared to the desired density of 1 × 10^3 ^to 1 × 10^7 ^yeasts/ml.

### Experimental protocol for *C. albicans *incubation with synovial fibroblasts

Dishes of synovial fibroblasts were placed in serum-free media (3 ml) overnight and then treated with either 200 μL PBS or 200 μL suspension of *C. albicans *(2 × 10^2 ^to 2 × 10^6 ^yeasts/dish) in 5% CO_2 _atmosphere at 37°C for 6 or 12 h. In some experiments synovial fibroblasts were pre-incubated with U0126 (Cell Signaling Technology, Beverly, MA, USA), a mitogen-activated protein kinase (MEK)1/2 inhibitor, at a concentration of 20 μM for 2 h; laminarin (Sigma) a β-glucan receptor blocking agent and specific inhibitor of dectin-1 activity at a concentration of 10 mg/ml for 1 h. MG-132 (Calbiochem, San Diego, CA, USA) as a NFκB inhibitor was co-incubated with synovial fibroblasts at a concentration of 35 μM. For the trans-well experiments (Transwell, Corning Incorporated, Corning, NY, USA), synovial fibroblasts were seeded in the upper chamber and *C. albicans *were plated in the lower chamber overnight, and then interacted for 12 h. In controls *C. albicans *were omitted from the lower chamber.

### Immunocytochemistry

After a 12-h co-culture of synovial fibroblasts and *C. albicans*, cells and fungi on dish were washed with ice-cold PBS twice and then fixed using 2 ml of a 1:1 methanol/acetone mixture per dish for 5 minutes at -20°C. Cells were then stained by immunocytochemistry. Immunodetection for COX-2 was performed with a standard avidin-biotin-peroxidase complex detection kit (DakoCytomation, Glostrup, Denmark). Dishes were washed twice with PBS and blocked by incubation with 200 μL 1% non-immune horse serum (Vector Laboratories, Burlingame, CA, USA) in 1% bovine serum albumin (BSA) in antibody diluent (DakoCytomation) for 30 minutes at room temperature. The solution was poured off and the cells incubated sequentially with anti-COX-2 epitope specific antibody (1:200) (Lab Vision Corporation, Cheshire, UK) or anti-phospho-ERK1/2 (1:100) (Cell Signaling) for 60 minutes, biotinylated secondary antibody (1:200) for 45 minutes, and horseradish peroxidase (HRP)-conjugated streptavidin for 20 minutes. Between each incubation cells were washed with Tris-buffered saline/Tween (TBST) (12.5 mM Tris/HCl, pH 7.6, 137 mM NaCl, 0.1% Tween 20) three times. The chromogen 3-amino-9-ethylcarbazole (AEC) was then added for 15 minutes and finally counterstained with Mayer's hematoxylin. The cells were mounted with a coverslip and visualized under light microscopy.

### Reverse transcription PCR

Total RNA was isolated from cells after a 12-h co-culture of synovial fibroblasts and *C. albicans *using easy-BLUE Total RNA Extraction Kit (iNtRON Biotechnology, Gyeonggi-do, Korea). For first strand cDNA synthesis, 3 μg of total RNA was used in a single-round RT reaction (total volume 20 μl), containing 0.75 μg oligo(dT)_14 _primer, 1 mM deoxynucleosides (dNTPs), 1 × first strand buffer, 0.4 mM dithiothreitol (DTT), 40 units RNaseOut recombinant ribonuclease inhibitor, and 200 units of superscript II reverse transcriptase (Gibco Invitrogen). The reverse transcription reaction was performed at 42°C for 2 h, followed by 95°C for 5 minutes. PCR was run using 0.9 μl of the reverse transcription reaction mixture as template, 0.4 mM of gene specific primers, 1 × PCR buffer, 0.25 mM dNTPs, and 1.5 units of Taq DNA polymerase (BioMan, Taipei, Taiwan). The amplification was carried out at 94°C for 1 minute, then for 30 cycles at 94°C for 1 minute, 56°C for 1 minute, and 72°C for 1 minute followed by a final extension at 72°C for 10 minutes. All PCR products were size-fractionated by a 1.5% agarose gel electrophoresis, and DNA bands were visualized by staining the gel with 0.1 μg/ml ethidium bromide. The bands were analyzed using gel documentation system (Bio-Profil, Bio-1D version 99; Viogene, Sunnyvale, CA, USA). The values were expressed as ratio of the band intensity of the target gene to glyceraldehyde-3-phosphate dehydrogenase (GAPDH) and the ratio of the band intensity of COX-2/GAPDH in the control condition was normalized to 1. Variance and *P *values were analyzed by Alphaimager 1220 V5.5 (Alpha Innotech Corporation, San Leandro, CA, USA). A Student t test was used for statistical comparison between groups. A *P *value of less than 0.05 was considered statistically significant.

The primers used were as follows: COX-2 5'-GTCTCTCATCTGCAATAATGTG-3' (sense) and 5'-ATCTGTGTGGGTACAAATTTG-3' (antisense) ([GenBank:S67722]; PCR product 801 base pairs (bp)); GAPDH 5'-CCCATCACCATCTTCCAGGAG-3' (sense) and 5'-GTTGTCATGGATGACCTTGGCC-3' (antisense) ([GenBank:X02231]; PCR product 284 bp).

### Analysis of COX-2, ERK1/2 and phospho-ERK1/2 expression

Following *C. albicans *infection cells for 12 h were immediately washed with ice-cold PBS containing 100 μM Na_3_VO_4 _(Sigma) and lysed *in situ *with ice-cold lysis buffer at 4°C for 15 minutes. Lysis buffer contained 1% Igepal (Sigma), 100 μM Na_3_VO_4_, and a protease inhibitor cocktail tablet (Roche Diagnostics, Mannheim, Germany). Whole cell lysates were collected after centrifugation at 14,500 rpm for 15 minutes. Protein concentration was determined by the Lowry method. Equal amounts of protein (20 μg) were loaded onto 10% SDS polyacrylamide gels and were transferred to polyvinylidene difluoride (PVDF) membranes (Millipore Immobilon-P, Sigma). Membranes were blocked overnight at 4°C with 2% BSA in TBST. After washing three times with TBST, blots were incubated for 1 h at room temperature with primary antibody (anti-COX-2, 1/1,000 dilution; anti-total ERK1/2, 1/2,000 dilution; anti-phospho-ERK1/2, 1/2,000 dilution) diluted with 2% BSA in TBST. After washing six times with TBST, the blots were then incubated with HRP-labeled secondary antibody (1/1,000 dilution) for 1 h at room temperature. Membranes were rewashed extensively and binding was detected using Enhanced Chemiluminescense western blotting detection system (Amersham Pharmacia Biotech, Piscataway, NJ, USA), according to the manufacturer's instructions. Anti-ERK1/2 and phospho-ERK1/2 antibodies were from Cell Signaling Technology. Mouse monoclonal antibody tubulin Ab-4 (primary antibody, 1/5,000 dilution; secondary antibody, 1/20,000 dilution) (Lab Vision) served as internal control. The band was semiquantified by densitometry using systems as described above.

### Activation of NFκB by electrophoretic-mobility shift assay (EMSA)

Cells were infected with 2 × 10^5 ^*C. albicans *at 37°C for 6 h. Nuclear and cytoplasmic extracts of synovial fibroblasts were prepared using NE-PER nuclear and cytoplasmic extraction reagents according to the manufacturer's protocols (Pierce, Rockford, IL, USA). A non-radioactive EMSA was performed using an EMSA kit according to the manufacturer's instructions (Panomics, Redwood City, CA, USA). Nuclear protein (8 μg) was used to bind biotinylated oligonucleotides containing the NFκB binding site for 30 minutes at room temperature. The blank control was nuclear extracts being replaced with water. A competition/cold control was set up by adding non-biotin-labeled cold probes to the reaction. Samples were separated in a non-denaturing polyacrylamide gel (6%, with 2.5% glycerol) and blotted on a Biodyne B Pre-cut Modified Nylon membrane (Pierce). The biotin was labeled with alkaline phosphatase-conjugated streptavidin and alkaline phosphatase was detected with Enhanced Chemiluminescense western blotting detection system (Amersham). The band was semiquantified by densitometry using systems as described above.

### Measurement of PGE_2_, IL1β, and TNFα production in culture medium

Cells were infected with 2 × 10^5 ^*C. albicans *in the presence or absence of U0126 (20 μM, pre-incubation for 2 h) at 37°C for 12 h. The culture supernatant was harvested, and PGE_2_, IL1β, and TNFα concentrations were measured by ELISA (R&D Systems, Minneapolis, MN, USA) according to the manufacturer's instructions.

## Results

### COX-2 induction by *C. albicans *infection

**Figure 1:**
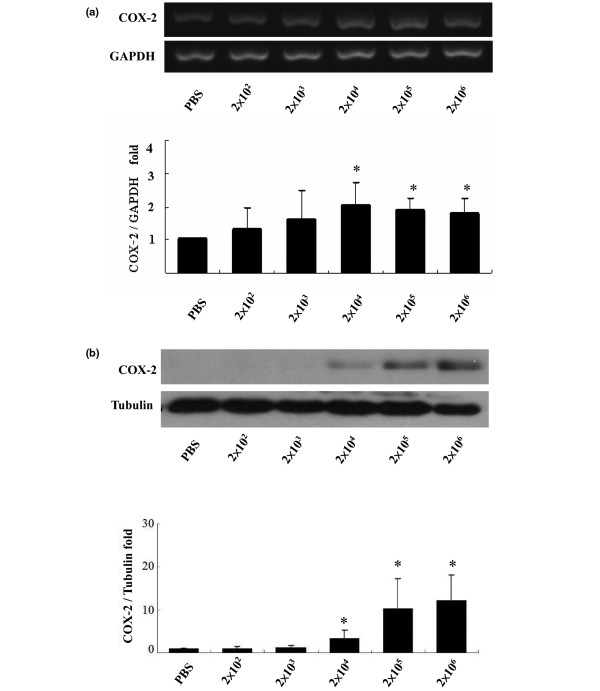
Cyclo-oxygenase 2 (COX-2) expression following co-culture of *Candida albicans *with synovial fibroblasts. COX-2 expression by synovial fibroblasts was assessed after 12-h co-culture of synovial fibroblasts with different seeding densities of *C. albicans*. **(a) **Gene expression. A representative agarose gel demonstrating COX-2 mRNA expression as assessed by reverse transcription polymerase chain reaction (RT-PCR). Glyceraldehyde 3-phosphate dehydrogenase (GAPDH) served as internal control. The graph shows the results of densitometric analysis of DNA bands expressed as the mean ± standard deviation (SD) of the relative fold change in COX-2/GAPDH ratio with the ratio of the control condition normalized to 1 (N = 6, * *P *< 0.05). **(b) **Protein expression. A representative western blot of COX-2 protein expression following infection by *C. albicans *with tubulin as an internal protein loading control. The graph shows the results of densitometric analysis of bands expressed as the mean ± SD of the relative change in COX-2/tubulin ratio with the ratio of the control condition normalized to 1 (N = 5, * *P *< 0.05).

The effect of *C. albicans *on COX-2 expression by synovial fibroblasts was assessed at the molecular and protein level. Extraction of total RNA from synovial fibroblasts was performed after 12-h co-culture of synovial fibroblasts with different seeding densities of *C. albicans *and COX-2 induction examined by RT-PCR. Addition of *C. albicans *to synovial fibroblasts increased COX-2 expression in a dose dependent manner. A significant increase in COX-2 expression over basal conditions was seen at a dose of 2 × 10^4 ^yeasts/dish (2.03 ± 0.74-fold increase, *P *= 0.0185) with no further increase when higher numbers of yeast were added (Figure 1a). The expression of COX-2 protein showed a similar pattern to that of mRNA expression (Figure 1b).

To ascertain whether COX-2 induction was mediated by production of a soluble mediator in the system culture medium was collected from co-cultures of synovial fibroblasts and *C. albicans *and added directly to non-infected synovial fibroblasts. No change in COX-2 expression was seen. The levels of IL1β and TNFα production were also undetectable (data not shown).

### ERK1/2 activation is necessary for *C. albicans *induction of COX-2 expression

**Figure 2:**
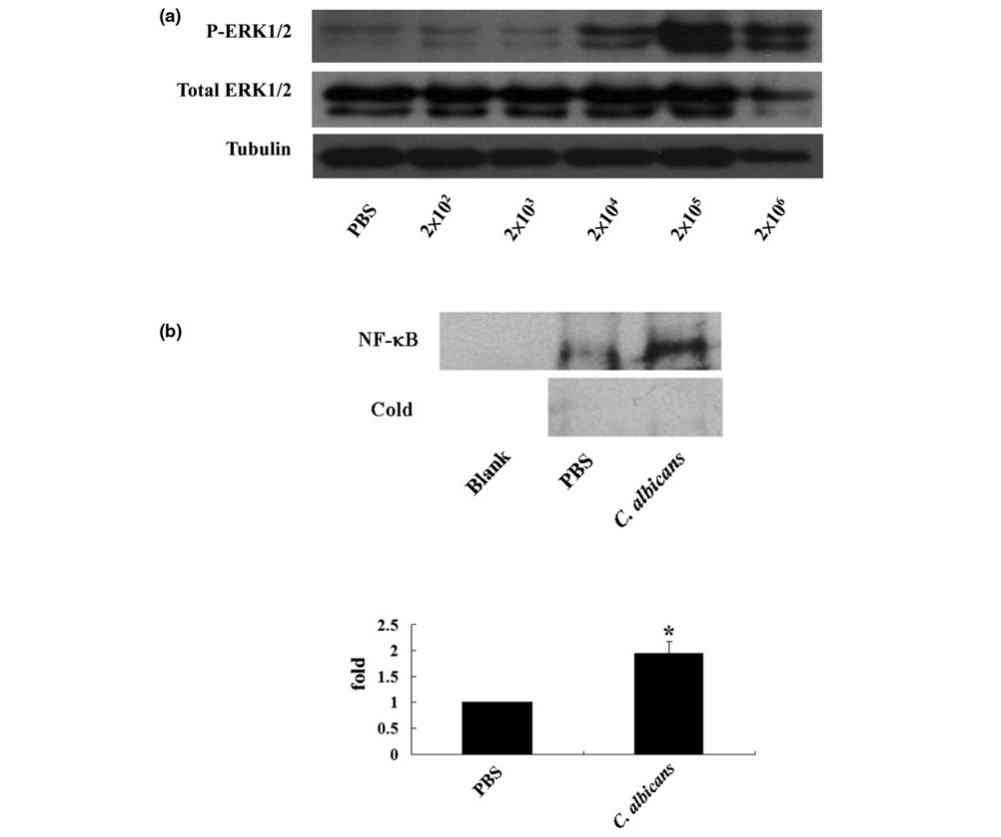
Activation of extracellular-regulated kinase (ERK1/2) and nuclear factor κB (NFκB) following infection of synovial fibroblasts with *Candida albicans*. **(a) **Synovial fibroblasts were infected for 12 h with *C. albicans *and levels of total and phosphorylated ERK1/2 (P-ERK1/2) assessed. Tubulin served as protein loading control. Shown is a representative western blot from one of five experiments. **(b) **Synovial fibroblasts were infected for 6 h with *C. albicans *and NFκB activation assessed by electrophoretic-mobility shift assay (EMSA). Upper panel: NFκB EMSA. The first lane of the NFκB series is the blank control. The lower series is the cold control. Lower panel: semiquantitative analysis of EMSA band density (N = 3, *P < 0.05).

COX-2 expression by proinflammatory cytokines is associated with ERK1/2 and NFκB activation. To establish if similar events were occurring with *C. albicans *infection of synovial fibroblasts a series of experiments were undertaken to identify whether either ERK1/2 or NFκB were activated under the experimental conditions that result in increased COX-2 expression. The results are shown in Figure 2. Co-incubation of synovial fibroblasts resulted in ERK1/2 activation in a dose dependent manner. Significant levels of ERK1/2 phosphorylation were identified with the addition of *C. albicans *at doses of 2 × 10^4 ^yeasts/dish and above (Figure 2a). Following co-culture of synovial fibroblasts with *C. albicans *at 2 × 10^5 ^yeasts/dish for 6 h, NFκB electrophoretic-mobility shift showed activation of NFκB (Figure 2b).

**Figure 3:**
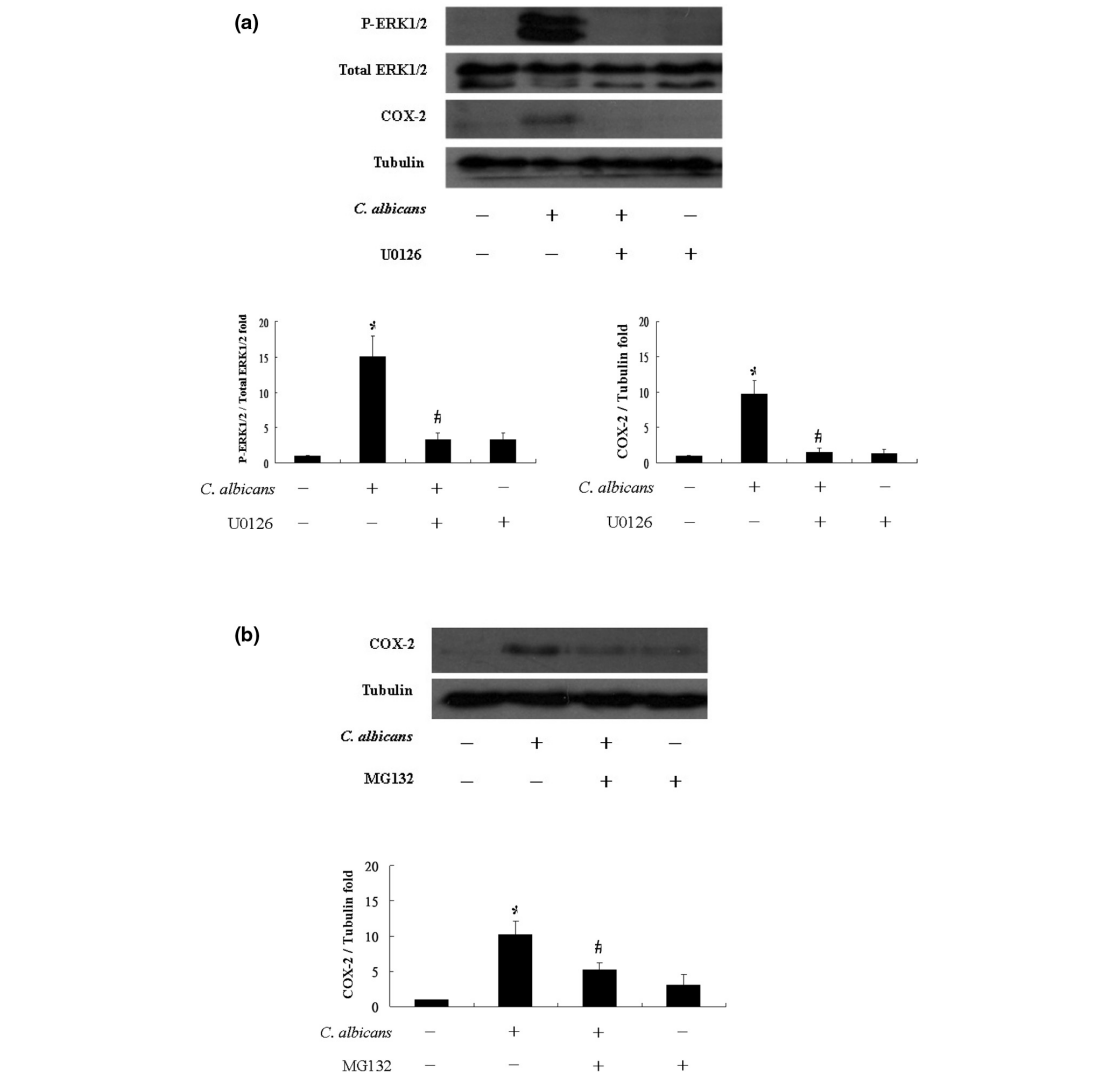
The effect of extracellular-regulated kinase (ERK) and nuclear factor κB (NFκB) inhibition on cyclo-oxygenase 2 (COX-2) production following infection of synovial fibroblasts with *Candida albicans*. Following infection of synovial fibroblasts for 12 h with 2 × 10^5 ^yeasts/dish in the absence or presence of **(a) **the mitogen-activated protein kinase (MEK)1/2 inhibitor U0126 or **(b) **the NFκB inhibitor MG-132 COX-2 protein levels were assessed by western blotting. Shown is a representative blots from N = 3 experiments. The graphs shows the results of densitometric analysis of bands expressed as the mean ± standard deviation (SD) of the relative fold change in band density with the ratio of the control condition normalized to 1 (N = 3, *, ^# ^*P *< 0.05).

**Figure 4:**
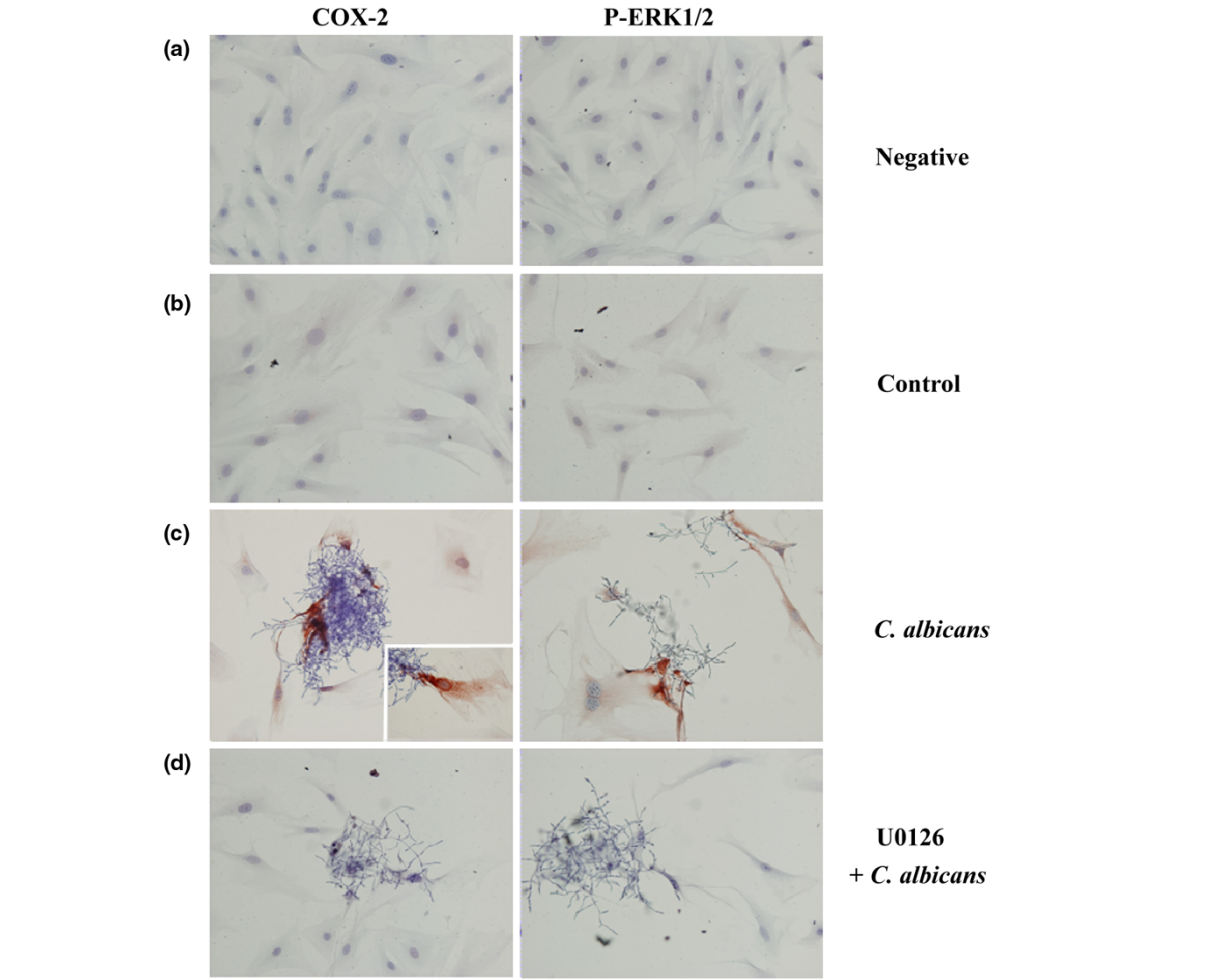
Immunocytochemical detection of cyclo-oxygenase 2 (COX-2) and phospho-extracellular-regulated kinase (ERK)1/2 expression in synovial fibroblasts infected with *Candida albicans *for 12 h. Synovial fibroblasts infected with *C. albicans *in the absence (c) or presence (d) of the mitogen-activated protein kinase (MEK)1/2 inhibitor U0126 were immunostained for COX-2 and phosphorylated ERK1/2. **(a) **Negative control with omitted primary antibody. **(b) **Control synovial fibroblasts. **(c) **Synovial fibroblasts infected with *C. albicans*. **(d) **Synovial fibroblasts infected with *C. albicans *in the presence of U0126 (insert × 1,000; others × 400).

We next examined whether COX-2 expression was regulated by ERK1/2 activation. Synovial fibroblasts were pretreated with U0126, a MEK1/2 inhibitor, at a concentration of 20 μM for 2 h before addition of *C. albicans *(2 × 10^5 ^yeasts/dish for 12 h) (Figure 3a). *C. albicans *increased ERK1/2 phosphorylation and COX-2 expression in the absence but not the presence of U0126. U0126 by itself had no effect on COX-2 expression or ERK1/2 phosphorylation. MG132 as an NFκB inhibitor suppressed the COX-2 expression (Figure 3b). Immunohistochemistry (Figure 4) demonstrates increased phospho-ERK1/2 and COX-2 expression in synovial fibroblasts to which *C. albicans *are adherent. However, the cells without *C. albicans *attachment demonstrated only very weak positivity. In the presence of U0126 no expression of phospho-ERK1/2 or COX-2 is demonstrable in the infected synovial fibroblasts.

### PGE_2 _production

**Figure 5:**
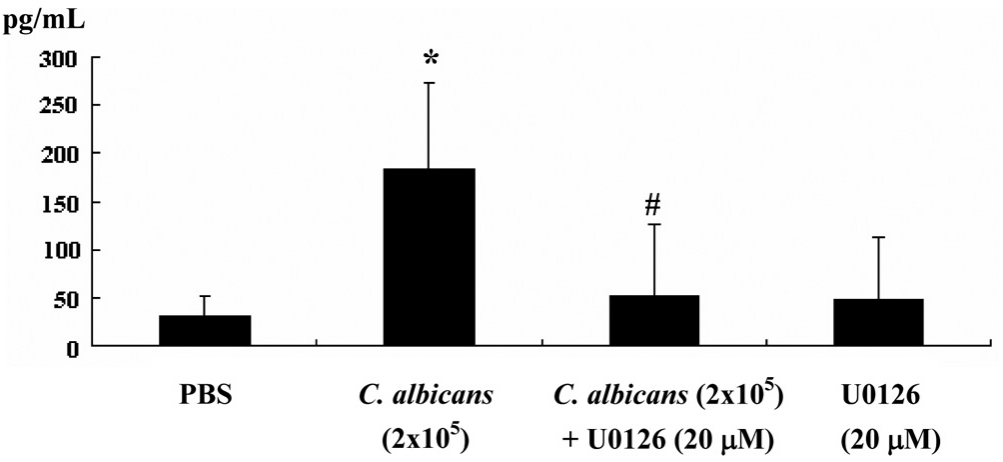
The effect of *Candida albicans *infection of prostaglandin E2 (PGE_2_) production by synovial fibroblasts. Synovial fibroblasts were infected with *C. albicans *2 × 10^5 ^yeasts/dish for 12 h in the absence or presence of mitogen-activated protein kinase (MEK1)/2 inhibitor U0126 and the supernatants were collected for assessment of PGE_2 _production by ELISA. N = 5, *C. albicans *vs phosphate-buffered saline (PBS), *P < 0.05; *C. albicans *+ U0126 vs *C. albicans *alone, #*P *< 0.05.

To assess whether increased expression of COX-2 was associated with changes in prostaglandin production levels of PGE_2 _released into the media was measured (Figure 5). In the presence of *C. albicans *infection PGE_2 _release into the media was significantly increased over basal levels. This effect of *C. albicans *was suppressed by the addition of U0126.

### Laminarin effect and trans-well experiment

**Figure 6:**
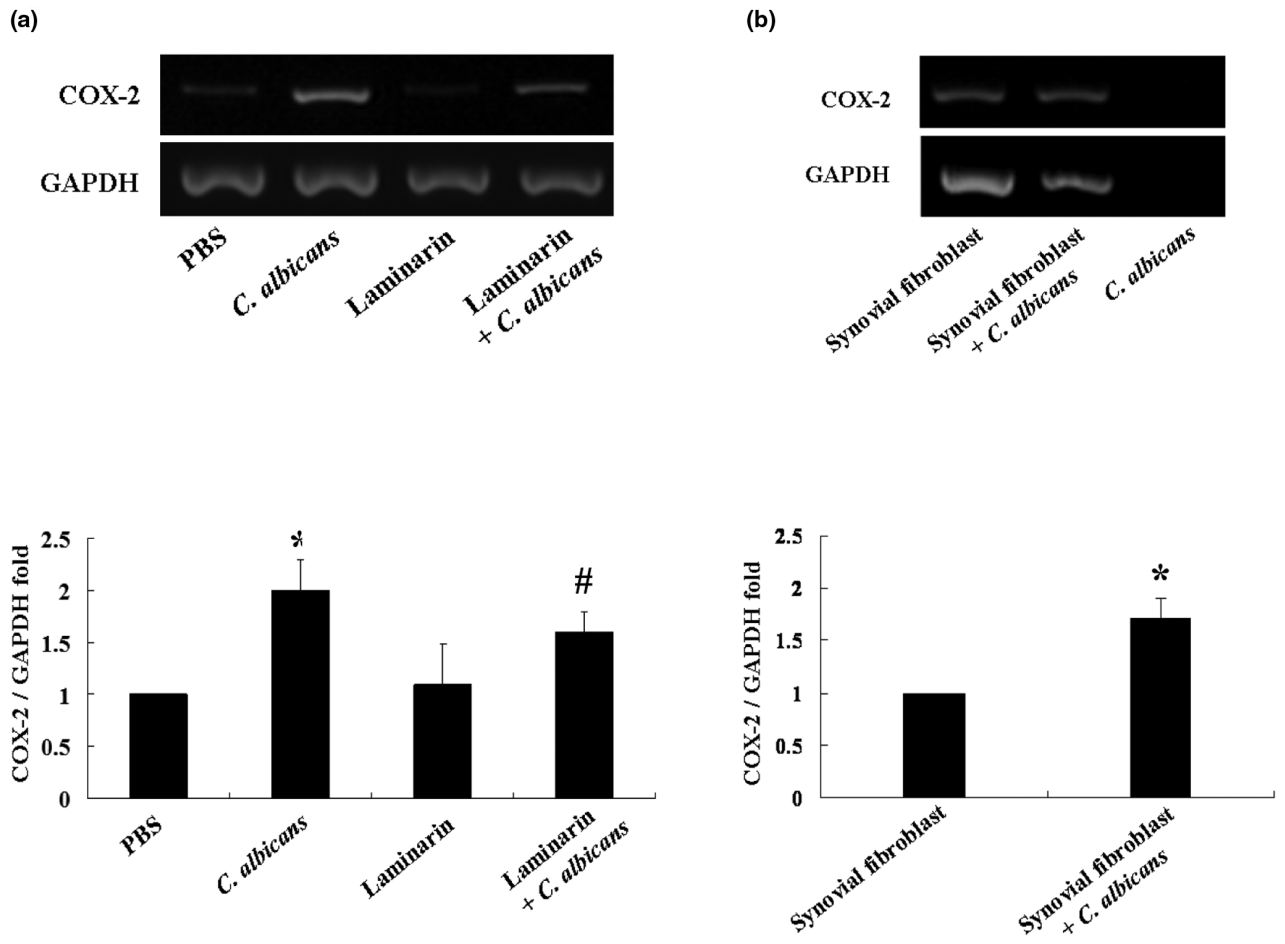
Requirement for dectin-1 and cell-cell interactions in upregulation of cyclo-oxygenase 2 (COX-2) expression (measured by RT-PCR) in synovial fibroblasts infected with *Candida albicans*. **(a) **Synovial fibroblasts were infected with *C. albicans *in the absence or presence of 10 mg/ml laminarin and COX-2 gene expression assessed. In the presence of *C. albicans *COX-2 gene expression by synovial fibroblasts is increased (*P < 0.05 *C. albicans *vs phosphate-buffered saline (PBS)). This increase in COX-2 gene expression is decreased by around 20% by laminarin (#*P *< 0.05 laminarin + *C. albicans *vs *C. albicans *alone). **(b) **Synovial fibroblasts and *C. albicans *were seeded in different chambers of trans-well plates overnight followed by 12 h of chamber interaction and subsequent assessment of COX-2 gene expression in synovial fibroblasts (lanes 1 and 2) and *C. albicans *(lane 3). Lane 1: synovial fibroblasts in the upper chamber and no *C. albicans *in the lower chamber. Lane 2: synovial fibroblasts in the upper chamber and *C. albicans *in the lower chamber. Lane 3: empty upper chamber and *C. albicans *in the lower chamber. RT-PCR using the RNA from *C. albicans *showed no band of COX-2 and glyceraldehyde-3-phosphate dehydrogenase (GAPDH) (N = 5, * *P *< 0.05).

To assess whether COX-2 induction was dependent on interactions with the dectin-1 receptor synovial fibroblasts were infected with *C. albicans *in the presence of laminarin (Figure 6). Laminarin had no effect on levels of synovial fibroblast COX-2 mRNA in the absence of *C. albicans*. Infection of synovial fibroblasts with *C. albicans *resulted in a 2 ± 0.3-fold increase in COX-2 gene expression (*P *< 0.05). In the presence of laminarin there was a lower, 1.6 + 0.3-fold, but significant (*P *< 0.05) increase in COX-2 gene expression when synovial fibroblasts were infected with *C. albicans*. This 20% decrease in COX-2 gene expression by laminarin was statistically significantly (*P *< 0.05, synovial fibroblasts + *C. albicans *vs synovial fibroblasts + *C. albicans *+ laminarin) (Figure 6a). COX-2 gene expression was significantly upregulated (1.71 ± 0.3-fold increase, *P *< 0.05) (Figure 6b) when synovial fibroblasts and *C. albicans *were co-cultured in different trans-well chambers. This indicates that direct contact may have only a minor contribution to the elevation of COX-2 gene expression seen when synovial fibroblasts are infected with *C. albicans*.

## Discussion

The present study has demonstrated that synovial fibroblast expression of COX-2, under the control of ERK1/2, is induced following *C. albicans *infection. Upregulation of COX-2 is associated with NFκB activation and appears to be more prominent in synovial fibroblasts adjacent to fungal colonies.

The finding that ERK1/2 phosphorylation occurs on exposure of synovial fibroblasts to *C. albicans *is consistent with observations of interactions of *C. albicans *with inflammatory and epithelial cells. Phagocytosis of *C. albicans *by macrophages results in ERK phosphorylation [29] and TNFα production by monocytes exposed to *C. albicans *is ERK dependent [32]. TLR2 appears to be the major receptor mediating PGE_2 _production by mouse macrophages in response to *C. albicans *[28]. *C. albicans *increases COX-2 expression in HeLa cells with roles for both TLR2 and TLR4 being identified [25]. Similar mechanisms are likely to be involved in the induction of COX-2 and PGE_2 _production in the current study.

Toll-like receptors have been shown to be involved in synovial inflammation in a wide range of inflammatory joint diseases including rheumatoid arthritis [34,35], Lyme arthritis [36], and streptococcal cell wall-induced arthritis [37]. TLR signaling is also likely to be involved in mediating proinflammatory responses and subsequent tissue destruction in fungal arthritis. The basic cell wall structure of *C. albicans *consists of a linear β-glucan backbone from which there are covalently attached branches of additional β-glucan and mannoproteins. Mannoproteins, highly antigenic proteins with large numbers of mannose groups attached have been shown to induce pro-inflammatory cytokine production in murine macrophages and human mononuclear cells [38]. Mannose-containing molecular patterns are also strong inducers of COX-2 expression and PGE_2 _production in human macrophages [39]. TLR have important roles in the induction of cytokines by fungi with TLR4 recognition of O-linked mannosyl residues present in the *C. albicans *cell wall are thought to be particularly important. Phospholipomannan, present in the cell surface of *C. albicans*, has been shown to be recognized by TLR2 [40]. Cytokine induction by *C. albicans *may also be through recognition of β-glucan by the dectin-1 (dentritic cell-associated C-type lectin-1)/TLR2 receptor complex [38].

Dectin-1, a major β-glucan receptor, has a number of antimicrobial functions in phagocytes including induction of cytokines and chemokines, possibly by collaborating with TLRs, involvement in endocytosis and phagocytosis and production of the respiratory burst [33]. Rat dectin-1 is involved in immune responses against fungi [41]. Laminarin, a soluble form of glucan blocks signaling through dectin-1 [42]. Laminarin decreases TNFα production by macrophages in response to zymosan and *C. albicans *infection [43]. In the current study laminarin partially blocked the increase in COX-2 mRNA that is seen when synovial fibroblasts are infected with *C. albicans*. This indicates that signaling through dectin-1 has a partial role in the upregulation of COX-2 gene expression. This may be through direct contact between *C. albicans *and synovial fibroblasts as the elevation of COX-2 gene expression was similar in trans-well experiments and experiments with laminarin where contact between *C. albicans *and synovial fibroblasts was possible.

## Conclusions

We show for the first time that COX-2 induction and PGE_2 _production occurs following infection of *C. albicans *to synovial fibroblasts and that this requires ERK1/2 activation and is associated with NFκB activation. These interactions may significantly contribute to the detrimental inflammatory/catabolic activities of synovial fibroblasts in septic arthritis induced by *C. albicans *and other fungi.

## Abbreviations

AEC: 3-amino-9-ethylcarbazole; BSA: bovine serum albumin; COX-2: cyclo-oxygenase 2; EDTA: ethylenediaminetetraacetic acid; ELISA: enzyme-linked immunosorbent assay; EMSA: electrophoretic-mobility shift assay; ERK: extracellular-regulated kinase; GAPDH: glyceraldehyde-3-phosphate dehydrogenase; HRP: horseradish peroxidase; IL: interleukin; JNK: c-Jun N-terminal kinase; MEK: mitogen-activated protein kinase; NFκB: nuclear factor κB; PBS: phosphate-buffered saline; PGE_2_: prostaglandin E_2_; PKC: protein kinase C; PVDF: polyvinylidene difluoride; RT-PCR: reverse transcription polymerase chain reaction; SD: Sprague-Dawley; TBST: Tris-buffered saline/Tween; TLR: Toll-like receptor; TNF: tumor necrosis factor.

## Competing interests

The authors declare that they have no competing interests.

## Authors' contributions

HSL conceived of the study, participated in its design and coordination, participated in the interpretation of results, and predominantly drafted the manuscript. CSL supervised the experiments by CJY. CJY carried out the RT-PCR, western blotting, EMSA, immunocytochemistry, and ELISA. SLS performed the statistical analysis. DMS helped to discuss the results and draft the manuscript. All authors read and approved the final manuscript.
